# Inter-individual relationships in empathic traits and feedback-related fronto-central brain activity: an event-related potential study

**DOI:** 10.1186/s40101-015-0053-7

**Published:** 2015-03-24

**Authors:** Yuki Motomura, Akira Takeshita, Yuka Egashira, Takayuki Nishimura, Yeon-kyu Kim, Shigeki Watanuki

**Affiliations:** Department of Psychophysiology, National Institute of Mental Health, National Center of Neurology and Psychiatry, 4-1-1 Ogawa-Higashi, Kodaira, Tokyo 187-8553 Japan; Japan Society for the Promotion of Science, 5-3-1 Kojimachi, Chiyoda-ku, Tokyo 102-0082 Japan; Integrative Brain Imaging Center, National Center of Neurology and Psychiatry, 4-1-1 Ogawa-Higashi, Kodaira, Tokyo 187-8553 Japan; Faculty of Design, Kyushu University, 4-9-1 Shiobarum, Minami-ku, Fukuoka 815-8540 Japan; Graduate School of Integrated Frontier Science, Kyushu University, 6-10-1 Hakozaki, Higashi-ku, Fukuoka 812-8581 Japan; Department of Public Health, Graduate School of Biomedical Sciences, Nagasaki University, 1-12-4 Sakamoto, Nagasaki, Japan

**Keywords:** Empathy, ERP, Feedback-related negativity, Error-related negativity, Error processing

## Abstract

**Background:**

Neuroimaging studies continue to indicate the major role the anterior cingulate cortex (ACC) plays in processing empathic responses. Error-related negativity (ERN), an event-related potential (ERP) thought to arise from the ACC, has been found to correlate with scores for individual empathic personality. This study investigated the relationship between empathic personality traits and the amplitude of feedback-related negativity (FRN), an ERP sourced from the ACC and similar to the ERN, using a task involving feedback of monetary gains or losses.

**Methods:**

Sixteen healthy participants answered an empathy trait questionnaire and performed a gambling task to elicit FRN. Because FRN amplitude is thought to be associated with attention, motivation, emotional state, and anxiety trait, we performed a partial correlation analysis between the empathic trait score and FRN amplitude while controlling for variables.

**Results:**

In partial correlation analysis, FRN amplitude was significantly inversely correlated with scores for personal distress and marginally correlated with scores for empathic concern and with total average score.

**Discussion:**

The study revealed for the first time an association between FRN and emotional empathic traits, after controlling for variables that can affect FRN amplitude. However, we also found a reversed directional correlation contrary to our expectations. This fronto-central brain activity may be associated with empathic properties via dopaminergic neuronal function. Future study using these electric potentials as experimental tools is expected to help elucidate the neurological mechanism of empathy.

## Background

Empathy is an important social mental function whereby individuals recognize and understand the ideas or emotions of others and experience the same emotional state as them [1]. Neuroimaging studies have consistently shown that the anterior cingulate cortex (ACC) plays a major role in processing empathic responses [2-4].

Because human empathic traits are genetically based [5] and present individual differences, it is thought there is some sort of physiological basis for them. Recent work has suggested that individual differences in empathic traits are associated with individual neurological characteristics (e.g., neuronal activity, sensitivity of neuronal response) in the ACC. Larson and colleagues reported that amplitude of error-related negativity (ERN), one of the components of event-related potential (ERP), correlated with scores for individual empathic personality [6]. ERN is a fronto-central negative potential elicited within 100 ms after an error response (e.g., incorrect button press) and is believed to be associated with conflict and cognitive control, including error processing [7,8]. ERN is thought to arise out of the ACC, which implies it shares at least a partial neurological basis with empathic function [9].

Another component related to error processing is feedback-related negativity (FRN), which is a negative component that occurs in the fronto-central region, as ERN does. FRN appears approximately 200 to 300 ms after error feedback presentation of executive tasks. It is calculated by subtracting the ERP waveform during presentation of a positive result (e.g., success and reward) from the waveform during presentation of a negative result (e.g., error and punishment) [10-13]. ERN is acquired by response-locked EEG averaging, while FRN is acquired by stimulus-locked averaging.

Both ERN and FRN are thought to arise from the dorsal ACC as an electric source [9,11,14]. Because these electric potentials have a similar appearance in error processing, Nieuwenhuis and colleagues hypothesized that they are associated with the same cognitive process [11]. In addition, FRN is sensitive to feedback from another person’s gambling task (even if the feedback is not personally useful) as well as to one’s own feedback, suggesting an implied connection with empathic function [10].

Given this background, we hypothesized that FRN amplitude predicts the variance of inter-individual empathic properties as well as ERN. This study investigated the relationship between empathic personality traits and amplitude of FRN with a task involving feedback of monetary gains or losses.

## Material and methods

### Ethics

This study was approved by the Ethics Committee of Kyushu University and was conducted in accordance with the Declaration of Helsinki.

### Participants

Participants were 16 healthy, right-handed university students (8 men, 8 women; mean age, 24.1 ± 3.32 years) who provided written informed consent prior to participating in the study. They were asked to obtain adequate sleep and refrain from intense exercise and alcohol intake the day before the experiment.

### Questionnaires

Participants came to the lab to answer questionnaires on anxiety and empathic traits, namely, the trait components of the State-Trait Anxiety Inventory (STAI) [15] and a multidimensional empathy scale for adolescents (MESA) [16], a Japanese questionnaire that is based on the interpersonal reactivity index (IRI) [17]. The STAI has two subscales: trait anxiety (STAI-trait) and state anxiety (STAI-state). The STAI has 40 items answered on a 4-point Likert scale ranging from ‘almost never’ to ‘almost always’. Each subscale is calculated by summing the scores (ranging from 1 to 4) of 20 different items. The MESA (IRI) has four subscales: (1) empathic concern, defined as a respondent’s tendency to experience feelings of warmth, compassion, and concern for others undergoing negative experiences; (2) personal distress, defined as a respondent’s experience of discomfort and anxiety when witnessing the negative experiences of others; (3) fantasy, defined as a respondent’s tendency to identify strongly with fictitious characters in books, movies, or plays; and (4) perspective taking, defined as a respondent’s tendency or ability to adopt the perspective or point of view of other people. In addition, we calculated the average score of these four subscales as a general empathic trait (total average). The MESA has 28 items answered on a 5-point Likert scale ranging from ‘does not describe me well’ to ‘describes me very well’. Each subscale is calculated by averaging the scores (ranging from 1 to 5) of 7 different items.

### Experimental protocol

**Figure 1:**
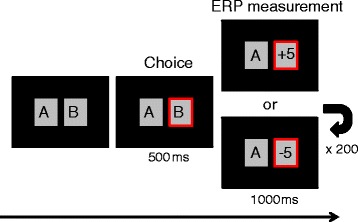
**Gambling task design.** Two boxes labeled A and B (angle of field, 2.62° × 3.59; interval between boxes, 2.76°) were displayed on the monitor. Each time the participant pressed a button to choose either A or B, a monetary gain or loss image was presented 500 ms after the choice on the monitor for 1000 ms. Each image (gain or loss) trial was performed 100 times, respectively, for a total of 200 images presented.

Participants individually performed a gambling task to elicit FRN [18]. Figure 1 shows the gambling task protocol. First, two boxes labeled A and B (angle of field, 2.62° × 3.59; interval between boxes, 2.76°) were displayed on a monitor. The participant chose either box A or B by pressing a button. Participants were informed beforehand that if they chose the wrong box, they would see an image showing their monetary loss (−5 JPY), and if they chose the correct box, they would see an image showing their monetary gain (+5 JPY). Each image was displayed 500 ms after pushing the button for 1,000 ms. They were also informed that they could obtain more or less reward money based on the total score presented in the task. Loss and gain images were actually presented an equal number of times in random order so that all participants received the same amount of reward. A trial for each feedback image (loss or gain) was performed 100 times, with 200 images presented in total. We averaged participants’ brain waves during presentation of loss or gain images and acquired time-locked ERPs. The task program script was coded using Windows Visual Basic 6.0 (Microsoft Corporation, Redmond, WA, USA) and stimuli were presented using the Multi Trigger System (MTS0410, Medical Try System, Tokyo, Japan) triggered by the task program. The monitor refresh rate was set to 75 Hz during task presentation and the response speed was 5 ms (LCD-A 173 KB-X, I/O DATA, Kanazawa, Japan).

### Subjective assessment

After the gambling task, participants rated how closely their feelings during the task matched four statements, using a 9-point scale from 1 (match) to 9 (not match): I concentrated on the task (concentration); I was interested in the task (interest); when a result was displayed, I paid attention to the result (attention); and I felt my emotions change by the result (emotional movement).

### ERP measurement and analysis

Electroencephalograms (EEGs) were acquired using a 64-channel net (HydroCel Geodesic Sensor Net; Electrical Geodesics, Inc., Eugene, OR, USA), amplified and measured (Net Amps 200 64-channel EEG Amplifier, Electrical Geodesics, Inc.; Net Station version 4.1.2, Electrical Geodesics, Inc.). Electrode resistance was maintained at <100 kΩ during the experiment, and data were continuously recorded at a sampling frequency of 250 Hz, with electrodes on Cz used as the system reference. The hardware band-pass filter was set at 0.1 to 100 Hz. EMSE-data editor version 5.2 (Source Signal Imaging Inc., La Mesa, CA, USA) was used for analysis. Measured EEGs were transformed using electrodes on the mastoid processes as the offline reference, and a software band-pass filter (0.1-30 Hz) was applied. Trials including artifacts above ±40 μV were rejected manually. Gain or loss image presentation for participants was set at 0 ms, and a −200- to 800-ms range was averaged to obtain the ERP waveform. Baseline correction of ERP was carried out by subtracting the mean value of −200 to 0 ms from the overall waveform. The number of additions to average was set at >60 times.

### Feedback-related negativity

**Figure 2:**
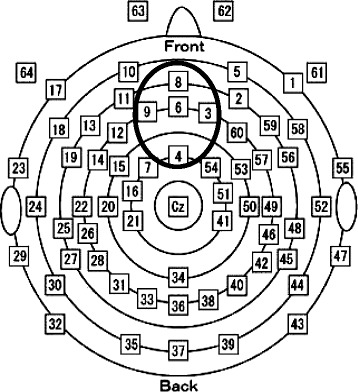
**Sensor layout and region of interest.** The distribution of each channel in the EGI 64 electrode HydroCel Geodesic Sensor Net is shown. The thick black circle indicates the fronto-central recording sites averaged for feedback-related negativity.

We calculated the mean amplitude of 200 to 300 ms in ERP of gain (+5 JPY) and loss (−5 JPY) trials. To minimize the effects of other positive ERP components overlapping with the FRN, many previous studies have examined subtracted waveforms (losses minus gains) [18-21]. In this study, the FRN value was calculated by subtracting values of gains from losses. We set the fronto-central region (channels 3, 4, 6, 8, and 9; Figure 2) as the region of interest for partial correlation analysis, and FRN values for these five channels were averaged.

### Statistical analysis

To confirm that FRN was elicited by the gambling task, we performed a paired *t* test to evaluate differences between the mean amplitude of 200 to 300 ms in the gain and loss trials. In addition, because FRN amplitude is thought to be associated with attention, motivation, emotional state, and anxiety trait [20,22-26], we controlled for the influence of those variables. Based on the technique Larson and colleagues used to examine the association between ERN amplitude and empathic traits [6], we performed partial correlation analysis (Spearman’s partial rank correlation) between the empathic trait score (MESA subscales: empathic concern, personal distress, fantasy, and perspective taking) and FRN amplitude with subjective assessment scores (concentration, interest, attention, emotional movement) and STAI-state and STAI-trait scores set as controlled variables. To normalize the effect of individual differences in ERP amplitude, we set the amplitude of 200 to 300 ms in the gain trial as a controlled variable. To consider multicollinearity among these controlled variables, we performed multiple regression analysis by the forced entry method, setting FRN amplitude as a dependent variable and these controlled variables for partial correlation analysis as independent variables. Because the variance inflation factor was over 10 for subjective assessment scores of attention and emotional movement, we removed these 2 indices from the partial correlation analysis. All statistical analyses were performed using SPSS PASW Statistics 18 software (IBM Japan Ltd., Tokyo, Japan), and differences were considered significant at *P* < 0.05.

## Results

### Demographics and subjective empathy

Table 1 shows data on participants’ anxiety state, anxiety trait, and empathic trait and results of the subjective empathy assessment.

**Table 1:** **Personality traits and subjective empathy assessment scores (** ***n*** **= 16)**

	**Mean (SD)**	
STAI-state	30.94	(7.71)
STAI-trait	40.50	(7.89)
MESA (IRI) empathic concern	3.68	(0.35)
MESA (IRI) personal distress	3.30	(0.81)
MESA (IRI) fantasy	3.42	(0.99)
MESA (IRI) perspective taking	3.01	(0.55)
MESA (IRI) total	3.35	(0.51)
Subjective assessment		
Concentration	3.75	(2.02)
Interest	4.19	(2.4)
Attention	3.44	(2.03)
Emotion	3.56	(2.25)

SD, standard deviation; STAI, State-Trait Anxiety Inventory; MESA, multidimensional empathy scale for adolescents; IRI, interpersonal reactivity index.

### ERP data

**Figure 3:**
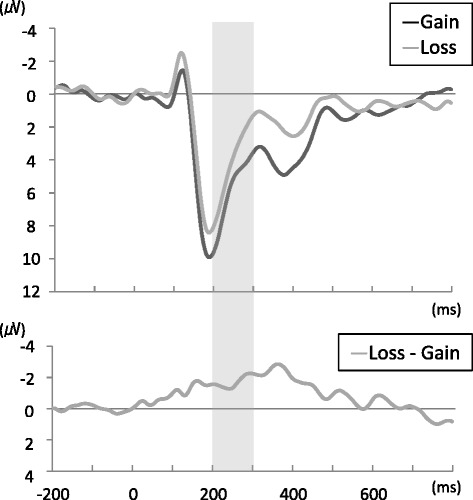
**Grand mean ERP waveform.** The upper graph shows the grand mean ERP waveform during presentation of gain and loss images, and the lower graph shows the subtracted (loss minus gain) ERP waveform. Gray-shaded areas represent the range of FRN. ERP, event-related potential; FRN, feedback-related negativity.

Figure 3 shows the grand mean ERP waveform during presentation of losses and gains and the subtracted waveform (loss minus gain) for fronto-central electrodes (channels 3, 4, 6, 8, and 9 averaged). Averaged amplitude differed significantly between the gain and loss conditions ([mean ± SD], gain 5.73 ± 3.29, loss 4.04 ± 2.41, loss minus gain −1.69 ± 2.13, *t* [15] = 3.17, *P* = 0.006).

### Partial correlation analysis

Table 2 shows results of the partial correlation analysis between empathic trait scores and FRN amplitude with subjective assessment scores, STAI-state and STAI-trait scores, and individual differences in ERP amplitude set as controlled variables. FRN amplitude was significantly positively correlated with scores for personal distress and marginally positively correlated with scores for empathic concern and with total average score. Because FRN is a negative electric potential, a positive correlation indicates that an individual with high empathic properties would exhibit small FRN amplitude.

**Table 2:** **Correlation between empathic traits and FRN amplitude, controlled for STAI, SA scores, and individual differences in ERP amplitude**

**Score**	***ρ***	***P***
Empathic concern	0.55^†^	0.081
Personal distress	0.67*	0.023
Fantasy	0.46	0.151
Perspective taking	0.002	0.996
Total average	0.58^†^	0.075

FRN, feedback-related negativity; STAI, State-Trait Anxiety Inventory; SA, subjective assessment. ^†^ *P* < 0.1; *P < 0.05; *df* = 9.

## Discussion

Partial correlation analysis between empathic trait scores and FRN amplitude, with subjective assessment scores, STAI-state and STAI-trait scores, and individual differences in ERP amplitude set as controlled variables, revealed that FRN amplitude was significantly inversely correlated with scores for personal distress and marginally correlated with scores for empathic concern and with total average score. According to Davis (1983), personal distress is defined as ‘the respondent experienc[ing] feelings of discomfort and anxiety when witnessing the negative experiences of others’, whereas empathic concern is defined as a ‘tendency for the respondent to experience feelings of warmth, compassion and concern for others undergoing negative experiences’. Empathic concern is often used synonymously with ‘sympathy’ and is categorized as an ‘emotional’ empathic trait, together with personal distress [27]. These emotional empathic traits are reported to predict subjective feelings of empathy [27], and they correlate significantly with physiological responses such as heart rate and skin conductance during the presentation of images eliciting empathy [28,29]. Thus, the present findings indicate that a highly emotional empathic individual would exhibit the neurological characteristic of small FRN amplitude.

As mentioned earlier, Larson and colleagues have reported that ERN amplitude correlated with scores of individual empathic personality [6]. Given that both ERN and FRN are thought to arise from the dorsal ACC as electric sources [9-11,14] and that FRN is sensitive to another person’s feedback (even if it is not personally useful) as well as to one’s own feedback [10], we expected FRN and ERN to have the same directional correlation with empathic traits, consistent with a shared relation to empathy based on the same underlying mechanism, which is thought to be related to empathic function through vigilance to one’s own performance and external environment or concern for positive outcomes [6]. Surprisingly, however, FRN exhibited a reversed directional correlation with empathic traits, which did not support Nieuwenhuis and colleagues’ hypothesis that FRN and ERN reflect the same neuronal process in the ACC [11]. Some studies have revealed differing characteristics between ERN and FRN. A study comparing healthy subjects with those with autism disorder [21,30,31], attention-deficit hyperactivity disorder, [32,33], or high obsessive-compulsive personality [14] reported different amplitudes for ERN and FRN. Although autism, attention-deficit hyperactivity disorder, and obsessive-compulsive disorder (OCD) are thought to be associated with the ACC, FRN and ERN might be differently influenced due to functional changes in the ACC resulting from these disorders. In particular, because high obsessive-compulsive individuals reportedly have higher ERN amplitudes, lower FRN amplitudes, [14], and high empathic traits [34], obsessive-compulsive personality might share the same neurological mechanism with empathic function. Holroyd and Coles hypothesized that FRN is related to the processing of conceptualizing negative feedback as ‘worse than expected outcomes’ or negative ‘prediction errors’, leading to an attenuation of phasic dopamine activity in the mesolimbic reward system (reinforcement learning theory [12]). Because OCD patients are known to have greater dopaminergic neuronal activity [35], attenuation of phasic dopamine activity might not occur as easily in OCD patients, who exhibit smaller FRN amplitudes. Some studies have indicated that dopaminergic activity is also related to empathic function, and they have shown the importance of dopamine receptors in the ACC for empathic-like activity in mice [36] and the relationship between empathic behavior and genetic polymorphisms of dopamine receptors [37] or dopamine beta-hydroxylase [38] in humans. Therefore, high dopaminergic activity might be associated with lower FRN amplitude of high empathic individuals as well as OCD. Future studies are needed to investigate this mechanism in more detail.

## Conclusion

This study revealed for the first time an association between FRN and emotional empathic traits after controlling for variables that can affect FRN amplitude. As we hypothesized, FRN amplitude correlated with individual empathic traits; however, we also found a reversed directional correlation contrary to our expectations. The response of dopaminergic neuronal function is similar to that of high obsessive-compulsive personality, suggesting a possible association with the relationship between fronto-central brain activity and empathic properties. Future study using this electric potential as an experimental tool is expected to contribute to elucidating the neurological mechanism of empathy.

## Abbreviations

ERP: event-related potential
fMRI: functional magnetic resonance imaging
FRN: feedback-related negativity
ERN: error-related negativity
ACC: anterior cingulate cortex
STAI: state-trait anxiety inventory
MESA: multidimensional empathy scale for adolescents
IRI: interpersonal reactivity index
